# Pilot Test of the Relationship and Financial Education TOGETHER Program for LGBTQ+ People in Relationships

**DOI:** 10.1111/jmft.70176

**Published:** 2026-10-01

**Authors:** S. D. Murry, Jessica N. Fish, Zeinab Azizi, Leah Young, Mariana K. Falconier

**Affiliations:** ^1^ Department of Family Science University of Maryland College Park Maryland USA; ^2^ Department of Human Development and Family Studies University of Illinois Urbana‐Champaign Champaign Illinois USA

**Keywords:** financial education, LGBTQ+ couples, program adaptation, relationships education

## Abstract

Financial stress negatively impacts health and relationships and relationship stress undermines financial management. LGBTQ+ individuals face additional stress from discrimination, yet no known programs address both relationship and financial stress for LGBTQ+ people. We pilot‐tested the adaptation of TOGETHER, an evidence‐based relationship and financial education program, for LGBTQ+ people in relationships by assessing its acceptability, appropriateness, and feasibility using mixed methods. We collected quantitative data on program satisfaction and usefulness through surveys completed anonymously by 31 participants and qualitative data through three focus groups with program participants (*N* = 12) and one focus group and six interviews with program staff (three case managers and 6 facilitators). Findings supported the adaptation's acceptability, appropriateness, and feasibility. Participants noted high program satisfaction and usefulness, but also offered ways in which the curriculum could be strengthened and even more focused on LGBTQ+ people in relationships

## 1 Introduction

LGBTQ+ Americans experience elevated chronic stress compared to their cisgender and heterosexual counterparts. These disparities arise from experiences with structural and interpersonal stigma, including prejudice, discrimination, harassment, and violence (i.e., minority stress; Meyer 2003), heightened familial and relationship stress (Thomeer et al. 2017), economic disadvantage, and financial stress (Deal et al. 2024). Financial stress is a comparatively understudied, pervasive form of chronic stress linked to broader structural inequities affecting LGBTQ+ Americans. LGBTQ+ Americans are less financially secure than their cisgender and heterosexual peers (Badgett et al. 2019) and are more likely to report lower household incomes, reduced savings, higher debt, and increased use of predatory lending (Delgadillo 2023). Transgender individuals and LGBTQ+ people of color (POC) experience compounded financial hardship that often affects their relational health and family stability (Badgett et al. 2019).

These economic inequities reflect the cumulative effects of structural discrimination across multiple domains. LGBTQ+ individuals experience documented inequities in hiring, wages, career advancement, and access to employer‐sponsored benefits. Family rejection or estrangement may further limit financial and social support (Badgett et al. 2021). Together, these structural barriers reduce opportunities to build an maintain economic stability over the life course. For transgender individuals and LGBTQ+ POC, multiple and intersecting forms of discrimination may further amplify these barriers, contributing to greater economic vulnerability and financial hardship (Badgett et al. 2019).

Financial stress is associated with adverse health outcomes and behavioral risk factors in the general population, including sleep disturbances (Lallukka et al. 2012), poor cardiovascular health (Swarup et al. 2024), coronary heart disease (Moran et al. 2019), smoking (Guillaumier et al. 2017), and mental health challenges (Richardson et al. 2013). For LGBTQ+ individuals, perceived financial stress is also associated with lower life satisfaction (Lazarevic et al. 2015). At the relational level, financial stress negatively affects couple functioning (for a review see Falconier and Jackson 2020) and interferes with relationship maintenance due to psychological stress and material constraints (Perez and Karney 2025). It is linked to increased relationship instability and dissatisfaction, greater frequency of negative partner interactions (e.g., conflict, psychological aggression), and reduced frequency of positive interactions (e.g., constructive conflict resolution, emotional support) (Falconier and Jackson 2020).

Just as financial stress impacts couple functioning, relationship quality also affects the quality of a couples' financial management (Peetz and Robson 2025). Despite the bidirectional effect, few couple relationship education (CRE) curricula include any financial content (Scott and Huz 2020), and only one CRE program centers financial education: The TOGETHER curriculum (Falconier 2015). TOGETHER is an evidence‐based psycho‐educational workshop (Falconier et al. 2022) for couples that integrates relationship and financial education with the goal of improving individual, relationship, and financial well‐being.

Despite the financial stressors experienced by LGBTQ+ participants, the few existing CREs developed for LGBTQ+ individuals have not centered finances, presenting a critical gap in both research and practice. CREs developed for LGBTQ+ participants include the evidence‐based curriculum Strengthening Same‐Sex Relationships (SSSR; Whitton et al. 2016), Better Together Online (Rostosky et al. 2025), 2GETHER, a CRE that integrates HIV prevention for male same‐sex couples (Macapagal et al. 2019), and the Couples Coping Enhancement Training – Sexual Minority Stress (CCET‐SMS; Randall et al. 2024), an adaptation designed to help LGBTQ+ participants cope with minority stress. Like many CRE models, these programs primarily focus on monogamous relationships which may limit their relevance for some LGBTQ+ individuals and relational structures (Scott et al. 2019). Further, because many of these programs are adaptations of existing interventions, questions remain regarding the extent to which adaptations can fully address the specific needs of LGBTQ+ populations compared to ground‐up developed programs (Chen et al. 2013). Given the need for LGBTQ+ CREs that focus on financial stress and well‐being, the TOGETHER program was adapted to serve LGBTQ+ participants. This paper provides an overview of that adaptation and presents findings from a pilot study evaluating its feasibility, acceptability, and appropriateness.

### 1.1 The TOGETHER Program

Unique among CREs (e.g., PREP; Markman et al. 2010; PREPARE‐ENRICH; Olson and Olson 2010; ELEVATE; Futris et al. 2014; Hold Me Tight; Johnson 2010), the TOGETHER curriculum was designed to improve relationship functioning as well as to reduce overall stress and financial stress and strengthen financial management (Falconier 2015). Because of its focus on financial stress, TOGETHER is particularly suited to the needs of economically disadvantaged couples (Arnold and Beelmann 2019). TOGETHER provides couples with knowledge and practical tools to strengthen their individual and dyadic stress management, communication, problem‐solving, and financial management.

The TOGETHER curriculum consists of nine modules that integrate conceptual learning with interactive skill‐building activities (e.g., role‐plays, games, take‐home exercises). Modules focus on: (1) the impact of financial stress and non‐financial stress on individuals and relationships; (2) individual stress management; (3) communication about stress; (4) dyadic stress management; (5) communication, de‐escalation, and emotion regulation during conversations about money and other sensitive topics; (6) partners' relationship with money and financial roles; (7) core financial management; (8) credit management; and (9) dyadic financial problem‐solving (for a detailed description of each module, see Falconier 2015).

The TOGETHER curriculum was originally developed as a 20‐h, 8 weekly two‐two‐and‐a‐half‐hour session in‐person workshop (Falconier 2015). Each TOGETHER workshop session is co‐facilitated by a relationship expert (e.g., couples' therapist) and a financial expert (e.g., financial counselor). From 2015 to 2025 the TOGETHER curriculum was offered alongside case management that connected couples to free or low‐cost community services and resources and was supported through a Healthy Marriage and Responsible Fatherhood grant awarded by the Administration for Children and Families, US Department of Health and Human Services.

#### 1.1.1 Effectiveness of the TOGETHER Program

After the initial pilot test (Falconier 2015), a randomized controlled trial (RCT) showed that participants who completed the 20‐h workshop experienced significant gains in managing financial stress and conflict, general and financial dyadic coping, and relationship satisfaction and commitment, compared to a no‐intervention control group. These improvements were maintained 6 months after program completion (Falconier et al. 2022). Later evaluations demonstrated the TOGETHER's program robust adaptability across varying durations, delivery formats, and cultural contexts. Evaluations of the 14‐h 6 week, one‐and‐a‐half‐hour (first session) and two‐and‐a‐half‐hour (second through sixth) session, in‐person curriculum (Falconier et al. 2023), online adaptation (Azizi et al. 2025), and JUNTOS en PAREJA–a linguistic and cultural adaptation for Latine immigrant couples–all provided evidence of maintaining the original's effectiveness (Yumiseva et al. 2024).

#### 1.1.2 The TOGETHER LGBTQ+ Adaptation

Given the success of previous TOGETHER adaptations and the established unmet need, the TOGETHER program was adapted to address the needs of LGBTQ+ participants. Consistent with available guidelines for CREs to inclusively and affirmatively serve LGBTQ+ individuals (Pasternack et al. 2023; Rivas‐Koehl et al. 2023), the adaptation involved changes to curriculum content, staff hiring and training, case management, and recruitment and outreach.

A team of LGBTQ+ and couple and family relationship experts reviewed the curriculum to make culturally tailored adjustments. Curriculum was revised to incorporate sexuality‐ and gender‐inclusive language and LGBTQ+‐specific content. New content included minority stress content, LGBTQ+‐specific financial considerations (e.g., paying for assisted reproductive technologies, adoption, gender affirming care) and using social supports (e.g., “chosen family”) to cope with stress. The program hired LGBTQ+‐identified facilitators and ensured that each staff member completed LGBTQ+‐specific cultural competency and LGBTQ+‐informed IPV training. Needs assessments were modified to include areas relevant to the LGBTQ+ community, and referral lists were updated to include verified LGBTQ+‐affirming resources. Community partnerships with LGBTQ+‐serving organizations were built and strengthened to support referrals and inform program changes. Marketing materials (e.g., flyers, newsletters, social media postings) and the website were revised to include LGBTQ+‐inclusive language, imagery, cultural symbols, and to celebrate LGBTQ+‐specific dates (e.g., National Coming Out Day). The first LGBTQ+‐specific workshops launched in June, coordinating with LGBTQ+ Pride Month.

### 1.2 The Present Study

Applying the Implementation Outcomes Framework (IOF; Proctor et al. 2011) the current study sought to pilot‐test the LGBTQ+ TOGETHER adaptation by examining its acceptability, appropriateness, and feasibility. Qualitative data from focus groups with participants and case managers, respectively, and interviews with facilitators were analyzed and integrated with quantitative program perception survey data. The study offers a model for future programmatic efforts to support LGBTQ+ relationships and financial well‐being.

## 2 Method

### 2.1 Participants

Program participants for LGBTQ+ TOGETHER were recruited from the Washington D.C. metropolitan area through several outreach methods: social media, advertisement, and community partner referrals. To participate in the workshops, participants had to be at least 18 years old, in a committed romantic relationship, and both partners had to be willing to participate. Participants were excluded if they reported psychiatric hospitalization in the past 12 months, an untreated substance use disorder, or safety concerns during the domestic violence screening.

All study participants took part in one of the first seven LGBTQ+ TOGETHER workshops, offered between June 2023 and March 2024. Workshops consisted of 6 weekly two‐and‐a‐half‐hour synchronous online sessions. Across these workshops, 30 couples (60 individuals) enrolled. Of the seven scheduled workshops, five were completed, one was cancelled after the first session, and another after the second session due to low attendance. Among the five completed workshops, 18 couples successfully finished all sessions, while six couples dropped out. Couples discontinued for several reasons, including scheduling conflicts, illness, and family emergencies. Notable discontinuations included one couple ending their relationship; one couple misunderstanding the workshop as group therapy; and one citing concerns about a non‐inclusive funding agency requiring assessments, the insufficient LGBTQ+ focus in the curriculum, and dissatisfaction with facilitators. All enrolled participants were invited to complete program evaluation questionnaires (PEQs) after their sixth and final workshop session, which were completed by 31 participants.

All participants, dropout participants, facilitators, and case managers from the first seven workshops were invited to take part in the present study's respective focus groups and interviews. Participants were invited if they attended at least five synchronous virtual sessions, or as dropout participants if they attended just one or two sessions. Only one partner per couple could participate in the same focus group to promote diverse perspectives and to minimize the potential for partners to influence one another's responses (Taylor and de Vocht 2011). Focus groups were unmixed, with participants and staff separated. Twelve participants (four per group) attended one of three participant focus groups, all three case managers participated in a single focus group, and all six facilitators participated in individual interviews. No focus groups were conducted with dropout participants as none responded to invitations.

In the participant focus groups, 41.7% identified as cisgender women, 33.3% as transgender or gender diverse (TGD), and 25% as cisgender men. For participants' sexual orientation, 58.3% identified as gay or lesbian and 41.7% as bisexual. Nine of the 12 participants (75%) identified as Black or African American and three (25%) as White. For age, 66.7% of participants were 21–34 years old and 33.3% were 35–54 years old. Half (50%) of the participants were married, half (50%) were employed full‐time, the majority (58.3%) reported a monthly income of less than $3000, and 75% reported having a bachelor's degree.

Interviewed facilitators identified as cisgender women (66.7%) or TGD (33.3%). For sexual orientation, 50% of facilitators identified as queer, 33.3% as bisexual, and 16.7% as heterosexual. In the case manager focus group, all identified as cisgender women and heterosexual. Respondents could select multiple gender identities and sexual orientations. Other demographic information was not collected.

### 2.2 Procedure

Quantitative data on program perception for the present study came from the anonymous PEQs that were completed at the end of the last workshop session, and from two items on program usefulness included from the survey required by the funding agency. For qualitative data, separate focus group recruitment emails were sent to program participants, case managers, and facilitators. Invitations included scheduled 1‐h focus group times and links to screening questionnaires to confirm eligibility. A separate focus group was offered to participants who attended two or fewer sessions before discontinuing the program, but no individuals expressed interest. Although facilitators were also invited to participate in a focus group, they instead participated in 1‐h individual interviews due to scheduling conflicts. Eligible and interested participants, case managers, and facilitators completed a consent form and a demographic questionnaire before participation. Each then received a Zoom link for their scheduled focus group or interview session. All focus groups and interviews were audio‐recorded and transcribed verbatim. Program participants that participated in focus groups were compensated with a $40 e‐gift card, and case managers and facilitators were compensated with paid work time. All study procedures were approved by the university's Institutional Review Board, and informed consent was obtained from all participants.

### 2.3 Measures

#### 2.3.1 Acceptability

Acceptability was defined as the perception among the program participants and staff (e.g., facilitators, case managers) that the LGBTQ+ adaptation was likable or satisfactory, and was assessed both quantitatively and qualitatively. In PEQs, acceptability items asked workshop participants to rate the extent to which the information presented was clear, well‐organized, and engaging, as well as their overall satisfaction with the workshop and likelihood of recommending the program to family and friends. Responses were measured on a 5‐point Likert‐type scale ranging from 1 = *strongly disagree* to 5 = *strongly agree*. Participants also evaluated the facilitators' performance using a 10‐point Likert‐type scale, ranging from 1 = *poor performance* to 10 = *excellent*. In focus groups, participants were asked open‐ended questions (e.g., “What were your overall impressions of the program?”; see Supporting Information: Table S1 for entire protocol) to assess their view of its acceptability. Staff's views were similarly assessed (e.g., “Is there anything else you'd like to share about your experience delivering the TOGETHER curriculum for LGBTQ+ participants?”) in the case manager focus group and facilitator interviews.

#### 2.3.2 Appropriateness

Appropriateness was defined as the relevance or compatibility of the adaptation to address the need for culturally competent stress management and financial education for the LGBTQ+ population and was assessed both quantitatively and qualitatively. In PEQs, appropriateness items asked workshop participants to evaluate the relevance of the workshop content; the usefulness of both relationship‐ and financial‐focused activities; whether others had noticed positive changes in their relationship as a result of their participation in the program; and to indicate their level of agreement with statements assessing whether they had learned communication, stress management, and financial management skills. All items used a five‐point Likert‐type scale ranging from 1 = *strongly disagree* to 5 = *strongly agree*. Two additional questions from the survey required by the funding agency asked participants to rate the overall helpfulness of the program (“overall, how helpful was the program to you?”) and program's helpfulness for financial well‐being (“overall, how helpful was the program for your financial well‐being?”) on a Likert‐type scale of 1 = *not helpful at all* to 5 = *extremely helpful*.

During interviews or focus groups, program participants were asked open‐ended questions about their perspective on the relevance of resources and materials and the cultural competence of TOGETHER staff (e.g., “In what ways did you think the sessions were relevant to your life experiences?”, “What was your overall impression of the TOGETHER facilitators and staff's awareness of and respect for people with LGBTQ+ identities?”). Similarly, staff were asked open‐ended questions about their perceptions of their own cultural competence and the adaptation's compatibility and relevance to LGBTQ+ participants (e.g., “In what ways did you attempt to make the workshop a safe and inclusive environment for LGBTQ+ participants?”, “What other topics related to relationship and financial education for LGBTQ+ participants should be included in the program's sessions, homework, or other?”).

#### 2.3.3 Feasibility

Feasibility is defined as the extent to which the adaptation was successful within the context of the TOGETHER program, including group composition and setting, and program implementation and delivery. Feasibility was quantitatively evaluated using program implementation indicators, including the number of facilitators trained and retained, workshop series launched and cancelled (and reasons), participant retention rate, session attendance (dosage), and adherence to curriculum. Curriculum adherence was evaluated through a Qualtrics survey completed by each facilitator at the end of every session. This survey included a question evaluating content completion (*1 = did not complete, 2 = partially completed or completed with changes, 3 = completed as outlined in the manual*), and a free‐text response question about the extent to which they had met the session's objectives and used the material and managed time effectively. It was also evaluated using two items from the PEQs that assessed the suitability of workshop time and location (1 = *strongly disagree* to 5 = *strongly agree)*. Qualitative insights were drawn from participant perspectives on how group size, compositions, and delivery modality influenced participation; and assessed among staff through one question in facilitators' interviews and case managers' focus groups (e.g., “In what ways do you think the program prepared you to deliver the curriculum for LGBTQ+ participants?”).

#### 2.3.4 Data Processes and Analyses

Given the pilot nature of the study, descriptive statistics were used to summarize participants' perceptions of workshop content and activities, facilitators' performance, and session attendance and dropout rates. Focus groups and interviews were conducted via Zoom. All participant focus groups were conducted by the first author, and staff focus groups and interviews were conducted by a single contracted interviewer who identifies as LGBTQ+ . Sessions were audio‐recorded and transcribed by Zoom's AI software with participant consent, then reviewed for accuracy, de‐identified, and uploaded and coded in the qualitative analysis software, Dedoose (2026). Thematic template analysis (Braun and Clarke 2006) was used to organize and interpret the data. Multiple LGBTQ+ and non‐LGBTQ+ coders reviewed transcripts to enhance credibility, resolving discrepancies through discussion until consensus. Representative quotes were selected to illustrate key themes and subthemes identified across participants' and staffs' perspectives.

An initial codebook was developed a priori based on IOF constructs of acceptability, appropriateness, and feasibility, alongside the interview guide, and refined throughout analysis. The coding framework supported alignment between interview prompts and analytic constructs. Following iterative application and refinement, the codebook was finalized upon achieving thematic saturation (Table 1).

**Table 1:** LGBTQ + TOGETHER codebook themes and subthemes.

Themes & Subthemes	Description
Acceptability	
Recruitment & Engagement	Likability of adaptation recruitment, enrollment, and engagement strategies.
Program Climate	Overall tone and inclusiveness of the adapted program environment.
Curriculum Acceptability	Satisfactoriness of the adaptation workshop materials, activities, and delivery.
Satisfaction With Staff	Likability, competence, and helpfulness of facilitators and case managers.
Appropriateness	
Curriculum Relevance	Usefulness and applicability of adaptation to LGBTQ+ participants' lives.
Program Resources	Usefulness of informational or referral resources offered.
Staff Cultural Competence	Staff's ability to deliver program to align with LGBTQ+ cultural values/experiences.
Feasibility	
Group Comp. & Setting	Effectiveness of group size, composition, and delivery modality.
Implementation & Delivery	Coordination of workshops, resources, and staff guidance during adaptation.

### 2.4 Validity

Researchers employed several strategies to enhance the validity of qualitative findings. Data were triangulated across participant and case manager focus groups and facilitator interviews to capture diverse viewpoints. Multiple coders independently reviewed, validated, and coded transcripts. Because positionality can inform data collection and analysis, the team engaged in ongoing reflexive discussion to evaluate their positionality in relation to the data. Discrepancies in coding were resolved through discussion to strengthen credibility. Member‐checking was not conducted to avoid placing additional burden on participants, but the use of an unaffiliated interviewer, verbatim transcription, and the inclusion of representative questions enhanced the trustworthiness and transparency of the findings.

## 3 Findings

Findings are organized according to the categories presented in Table 1. Within each section, quantitative results are presented first followed by qualitative findings.

### 3.1 Acceptability

In PEQs, participants reported high average ratings of satisfaction with the overall LGBTQ+ TOGETHER workshop (*M* = 4.71, SD = 0.16), and found curriculum content to be highly clear, organized, and engaging with means ranging from 4.54 to 4.69 (see Table 2). They also strongly agreed with recommending the program to family and friends (*M* = 4.64, SD = 0.29) and ranked the couple facilitator's (*M* = 9.66, SD = 0.07) and the financial facilitator's (*M* = 9.76, SD = 0.10) performance very highly.

**Table 2:** Program evaluation questionnaire (*n* = 31).

Items		Mean	SD
Acceptability	The information presented was clear.	4.69	0.16
	The information presented was well‐organized.	4.68	0.18
	The information presented was engaging.	4.54	0.17
	I would recommend the TOGETHER workshop to my friends or family.	4.64	0.29
	Overall, I am satisfied with the TOGETHER workshop.	4.71	0.16
	How would you rate the performance of the couple facilitator?	9.66	0.07
	How would you rate the performance of the financial facilitator?	9.76	0.10
Appropriateness	The information presented was relevant to my family situation.	4.62	0.23
	The activities for healthy relationships were appropriate.	4.77	0.12
	The activities for healthy relationships were useful.	4.73	0.15
	The activities for financial education were appropriate.	4.77	0.09
	The activities for financial education were useful.	4.71	0.11
	As a result of my participation in the program, I have learned skills that I…		
	plan to implement into my communication.	4.66	0.18
	plan to implement into my stress management.	4.54	0.37
	plan to implement into my financial management.	4.66	0.24
	As a result, others notice a positive change in us as a couple.	4.16	0.24
Feasibility	The sessions were offered at a time that worked well for me.	4.42	0.35
	The sessions were offered at a location that worked well for me.	4.85	0.10

#### 3.1.1 Participant Recruitment and Engagement

Participants reported feeling drawn to the program because it was marketed as LGBTQ+‐specific. One participant described the materials by saying, “There are markers to kind of grab someone's eye, depending on their identity. So, like, if you add any bright colors, the flag or rainbow, you know, it's gonna grab my attention as a part of the community.” Another participant shared that the materials “attracted me because I've had some bad experiences with different therapists that were [not affirming] so I was like, ‘Oh, finally something for us.” Similarly, TOGETHER's partnering with LGBTQ+ community organizations encouraged workshop enrollment. As one participant emphasized, “[An LGBTQ+ community partner was] advertising it. So, I knew that it's coming from a credible entity.” Participants also discussed the community‐centric appeal of the LGBTQ+‐specific group format: “We were looking for more LGBT community…I thought, ‘It's a workshop, so, there should be other couples in the area that we can maybe start to build community with… while still working towards [workshop goals].’” And “most attractive to me was the fact that it was LGBTQ+ , [so it] created that safe space where I knew I was gonna be around other people who had similar backgrounds.” These findings suggest that the adaptation's LGBTQ+‐specific branding and partnerships signaled workshop safety, credibility, and community. Case managers and facilitators were not asked about recruitment and engagement because they were not directly involved in recruitment activities.

#### 3.1.2 Program Climate

Participants described the program climate as a particular strength of the intervention, emphasizing that LGBTQ+ representation among participants and facilitators created an affirming and safe environment that supported open discussion and connection to community. One participant reflected on the experience of being among others with a diverse range of sexual and gender identities: “Seeing all of the… different elements along [that] gradient of queer identity…was really affirming for me.” Participants emphasized they felt safe in the workshop, as one explained, “Knowing that we had a safe space to discuss what we needed or ask the questions that maybe we felt like we couldn't ask in other settings was nice.” Participants also highlighted the supportive and interactive group dynamic, with one noting, “We had great facilitators…[who were] kind of letting you speak… and even the other participants were great as well, going back and forth and piggybacking off each other and giving advice.” Staff perspectives reinforced these experiences, with facilitators describing intentionally fostering safety to be affirming and supportive. As a facilitator shared, “The biggest strength of the LGBTQ+ group [was] that people were bonding with each other so tightly. They were talking about creating a book group or even meeting up outside of the [workshop], which I have never experienced in another group that I've led.”

#### 3.1.3 Curriculum Acceptability

Participants discussed their appreciation for the LGBTQ+‐tailored curriculum content. As a participant explained, “I really like the LGBTQ+ component of [the program], because just being an underserved community, there really aren't too many safe spaces out there that are specifically targeted for [us].” Another participant, while reflecting on the relative absence of cisgender and heterosexist relationship assumptions in the curriculum, said, “the way [my partner and I] divide our funds… does not reflect… these assumptions. Diving into content [that reflected] that and having these uncomfortable, but comfortable conversations was great.”

Participants also appreciated the program's focus on both relationship and financial topics. As one said, “I did love the fact that other things were offered [alongside the financial component].” Although participants offered positive feedback, facilitator perspectives introduced a more critical view of curriculum acceptability. One facilitator believed that participants “are really just not connecting with the program at all because [of] … not feeling comfortable with some topics, like family… They've mentioned not everyone has a family, can make a family.” Another facilitator shared, “I think that there have been some moments where I, myself, or participants have had a reaction to the curriculum because … it's pretty palpable that it wasn't designed by and for queer and trans people.” Notably, participants did not mirror facilitator concerns.

#### 3.1.4 Satisfaction With Staff

Participants were satisfied with the staff's allyship, noting that facilitators were “very affirming…welcoming…encouraging and supportive.” Participants felt that “you could tell that [facilitators] were there for us and wanted to be there,” many emphasizing the staff's efforts to establish personal connections by “sharing their real‐life experiences” as LGBTQ+ people and by getting to know program participants as individuals. Even if it was unclear whether a staff member identified as LGBTQ+ , they “seemed like they were allies.” Staff perspectives were consistent with these experiences, with facilitators also describing positive perceptions of their colleagues. Facilitators emphasized that their shared LGBTQ+ identity allowed them to, “share in [participants’] backstory,” which helped participants “feel more comfortable…and go, ‘I am in the right place.’” Although all focus group responses were positive, one couple who discontinued the workshop cited staff dissatisfaction and did not respond to focus group requests.

### 3.2 Appropriateness

Participants reported high levels of program appropriateness in the SEQs, with ratings of applicability and usefulness of workshop content and activities ranging from 4.62 to 4.77. They indicated that they learned skills they plan to apply in communication (*M* = 4.66, SD = 0.18), stress management (*M* = 4.54, SD = 0.37), and financial management (*M* = 4.66, SD = 0.24). Participants reported that their participation in the program led to observable positive changes in their relationship (*M* = 4.16, SD = 0.24). Participants also reported high levels of overall program helpfulness (*M* = 4.40, SD = 0.81) and financial well‐being helpfulness (*M* = 4.40, SD = 0.84).

#### 3.2.1 Curriculum Relevance

Workshop participants and staff agreed that the curriculum was highly relevant and applicable to participants' relational and financial experiences and needs. Participants emphasized the particular relevance of integrating financial and relational content, describing it as directly applicable to their lives. As one participant shared, “We were going through some financial hardships… all the content was very relevant… I felt like it was very spot on.”

Part of this perceived relevance is attributed to the curriculum's lack of cissexist or heteronormative gender assumptions. Participants highlighted the curriculum's inclusive and gender‐neutral framing as supporting its fit across diverse relationships. As one explained, “I don't think I saw any gendered terminology in the material… I just felt like this is a program targeted towards couples and at this moment they're just targeting towards LGBT couples.” Others emphasized how this lack of assumed roles contributed to a sense of equity and flexibility in the content. One participant noted the curriculum's applicability across relationships types: “There was potential for whatever kind of gender dynamic [because the content] was gender neutral … I never remember feeling any kind of ostracization from the material at all.” Facilitators echoed this sentiment saying “I think [the whole curriculum] relates” and “is excellent as a blanket for any groups.”

Participants further emphasized the particular relevance of the financial component, often describing it as a primary reason for enrollment. As one participant shared: “I liked the money management aspects … because it has been something that's been, in my relationship, [causing some issues] and when I saw the program, I just had to enroll.” While acknowledging the relevance of financial content, facilitators emphasized that the curriculum could be more explicitly tailored to reflect LGBTQ+ lived realities. One facilitator highlighted the importance of the financial content given structural barriers, noting that, “Financial difficulties [are] one of the biggest barriers to marriage and family building.” Facilitators identified gaps in how family structures were represented, explaining that “there's a lot of talk about family and the way that the family structure works. And that is something that is or can be fraught for members of the LGBTQ community. You know, they don't have the same safety nets many straight people have.” Notably, participants did not mirror these sentiments.

#### 3.2.2 Program Resources

Participants and staff described case management services and financial counseling as important supports, with participants emphasizing the value of LGBTQ+‐specific resources (e.g., “I did love … [the] wrap around service”). Participants highlighted the importance of affirming and relevant resources as part of the program. However, participants also identified limitations in the fit and applicability of available resources, noting that resources were not always relevant or available. For example, one participant said the resources “weren't super relevant… it didn't end up being stuff that I could really use.” Another described independently seeking out a LGBTQ+‐identified financial planner outside of providing resources, explaining, “I researched and I found a queer couple that runs a financial business… so maybe some resources like that [would be helpful].” Staff perspectives were consistent with these experiences, as case managers reportedly took considerable effort to expand resource list offerings as part of the adaptation, but acknowledged limitations. One case manager noted, “We have a lot of resources, but sometimes not enough for [LGBTQ+ participants]… you need to go a little bit more in depth with the resources.”

#### 3.2.3 Staff Cultural Competence

Participants described multiple ways staff's cultural competence shaped the appropriateness of the adaptation's content and delivery. Participants described facilitators as being affirming. One participant emphasized that workshop facilitators' “terminology, demeanor and response and tone and delivery… all of that was affirming for me personally.” Participants also noted how facilitators supplemented the curriculum and discussion using cultural knowledge: “the facilitators would [say], ‘This is a little different for queer folks… we don't have these kinds of defined gender roles in relationships.’ It wasn't explicitly in the content or slides, but more just as we were talking about things.” This extension of content through discussion helped align the curriculum with participants' lived experiences. Facilitators similarly described adapting materials in response to participant needs. For example, one facilitator recalled modifying a body scan activity to support a transgender participant, explaining that “One person had just received top surgery and… had a really difficult time engaging in that because of the active pain… So that exercise, although it is meant to be relaxation… was actually triggering for them.”

Participants consistently emphasized that the facilitators' LGBTQ+ identities and lived experiences supported workshop relevance. One participant shared, “I think the facilitators and staff really did an amazing job of connecting with us, just period. But also, being knowledgeable, sharing real real‐life experiences themselves.” Another noted the value of facilitators' LGBTQ+ relationship experiences, explaining, “One of them was in a same‐gender‐loving (SGL) relationship, so they were able to [share] some of [their relationship] experiences. That made it feel safer… knowing that the person who is talking to us is also living some of the same things.” Facilitators also observed the identity alignment as meaningful. A facilitator said, “There is a level of lived experience that is really, really helpful.” Although case managers did not identify as LGBTQ+ , participants emphasized that “everyone was very inclusive of our community.” Importantly, case managers described the training as supporting their cultural competence. One explained, “I learned so much that I didn't know … the trainings definitely did help.”

#### 3.2.4 Recommendations to Enhance Appropriateness

Participants and facilitators suggested ways to improve the program content. For example, a participant suggested including content about “specific stressors LGBT folks face, [such as] difficulty with family and family support and job discrimination… And how the systems aren't always set up for queer folks.” Comments from facilitators echoed this suggestion: “There needs to be additions to the program that are more specific [to the] discrimination that queer people face when interfacing with financial institutions, and specific stressors that occur around identity and financial stress.” Facilitators also suggested “queering the family structure” in the curriculum by adding content that reflects the breadth of LGBTQ+ family structures.

Participants and staff emphasized the need for greater racial diversity among facilitators and adjusting recruitment and curriculum language to be more racially inclusive. One participant suggested “having [workshops] be multiracial facilitated.” Another participant suggested expanding program language to include the term same‐gender‐loving: “Especially for POC, black, people I know, SGL is more and more of a term… that has become more prominent.”

Participants and facilitators also specifically mentioned adapting the program to be more inclusive of individuals identifying as disabled and neurodivergent. For instance, a participant suggested, “It could be helpful to have a group geared towards folks who are disabled. Queer disabled comes with so much financial stress.” Facilitators suggested “making things friendly for neurodivergent folks, as the LGBTQ population has a much higher rate of neurodiversity” and adding “neurodiverse recommendations where we could” in workshop activities.

### 3.3 Feasibility

Participants reported high levels of agreement that the workshop sessions were offered at a suitable time (*M* = 4.42, SD = 0.35) and location (*M* = 4.85, SD = 0.10). Participants completing the workshop attended an average of 5.86 sessions (SD = 0.42). Across the completed workshops, six out of 24 couples (25%) dropped out of the program due to mismatch of program expectations, family or medical emergencies, or scheduling conflicts. Of those completing the workshop, 32 participants attended all six sessions, three attended five sessions, and one attended four. Seven LGBTQ+‐identified facilitators were trained, and six retained. All curriculum adherence surveys were completed and responses indicated 98.7% adherence.

#### 3.3.1 Group Composition and Setting

Participants described ways that the group composition and setting supported program participation, particularly highlighting the accessibility of the online format and LGBTQ+‐ specific group settings. For example, one participant said, “It being virtual was really helpful. It felt like I could access it much easier than going into class,” emphasizing how the delivery format reduced barriers to participation. Participants and facilitators also described the LGBTQ+‐specific group composition as facilitating engagement and participation by creating a space that felt accessible and relevant. One participant praised the adaptation for “[going] above and beyond to try to hire members from the community” to create the LGBTQ+‐specific settings. Another “[liked] having other couples that identify as LGBTQ” in the workshop. Another elaborated, “I think creating and carving out that space was done successfully and is a successful move on the part of the TOGETHER program.” A facilitator agreed that LGBTQ+ participants “having their own space, our own space, that's carved out where we can be in community with other queers is always important.”

#### 3.3.2 Program Implementation and Delivery

Because participants were not involved in implementation, this section reflects staff perspectives. Staff described the adaptation's implementation and delivery as feasible, with training, ongoing supervision, and team collaboration supporting delivery. Case managers noted that the cultural competency trainings were particularly helpful for preparing them to deliver the adaptation. One case manager explained, “I did learn so much that I didn't know, also just how to be careful and saying certain things and all those important things.” Ongoing team collaboration further supported implementation through regular meetings where staff could address emerging challenges and refine delivery. A facilitator shared, “What was most helpful, [since we] were the first ones running the LGBTQ group, [we] would meet with the team every week to discuss issues that were arising.” A case manager echoed this, saying, “We always communicate about the feedback the participants are giving… because we want the workshop to get better,” indicating continuous feedback supported responsive changes during implementation. At the same time, staff identified that the primary implementation and delivery challenge was recruiting enough LGBTQ+‐identified facilitators, especially with racially and ethnically diverse identities, to meet the demand for the workshops. As one facilitator described: “When we [need] subs, we try our best to get [one] within the LGBTQ, but when we can't, we just [pick] a facilitator that can just sub that day. So definitely just having LGBTQ subs … could make a big difference.”

#### 3.3.3 Future Feasible Adaptations

When asked about offering workshops to different LGBTQ+ subgroups, participants endorsed the idea while stating that all LGBTQ+ people should be welcome to participate in the general LGBTQ+ workshop. A participant explained, “I think having everyone in one group is beneficial, but if someone wants to further experience [a workshop] with people who have more [similar identities] could also be helpful.” Multiple participants recommended offering a TOGETHER workshop specifically for TGD or consensually non monogamous (CNM) participants. When talking about these options, one participant said the offerings would be beneficial “because when you look at [transgender individuals'] lifestyles, sometimes how they end up having to obtain money is different. And then also when you look at poly lifestyles, how they sometimes end up having [and] handling budgets for … 4 or 5 individuals in one household.”

## 4 Discussion

This study investigated the acceptability, appropriateness, and feasibility of the LGBTQ+ TOGETHER adaptation, a 14‐h, six‐session, relationship and financial stress CRE delivered via Zoom. Overall, LGBTQ+ TOGETHER participants and staff found the program to be acceptable, appropriate, and feasible. To our knowledge, no other CREs currently address the interaction of relationship and financial well‐being for LGBTQ+ populations, positioning this model as a novel contribution. Regarding acceptability, findings were supported by both quantitative and qualitative data, and were consistent with previous TOGETHER evaluations (Falconier et al. 2022, 2023; Azizi et al. 2025). Participants and staff described the curriculum as clear, well‐organized, and engaging, particularly highlighting the usefulness of financial management strategies. Participants also valued the LGBTQ+‐targeted recruitment strategy for its culturally‐relevant advertisements, collaboration with LGBTQ+ community organizations, and offer of financial management education (Hatch et al. 2020). The LGBTQ+‐exclusive format and staff's affirming practices fostered a safe environment. Together, these findings indicate that recruitment strategies, program climate, curriculum content, and staff's skill contributed to positive experiences and enhanced engagement by reducing barriers related to mistrust and perceived irrelevance in CREs (Whitton et al. 2023). At the same time, participants and staff identified areas where greater LGBTQ+‐specific tailoring was needed.

Regarding appropriateness, findings were supported by both participant and staff perspectives, indicating that the program was relevant and useful for LGBTQ+ participants. High PEQ ratings, particularly for overall and financial helpfulness, reflected participants' perceived value of the relationship and financial management content. Participants and staff agreed the curriculum was broadly inclusive and largely free of cisgender and heterosexist bias, suggesting improved fit. However, both groups emphasized the need for additional LGBTQ+‐specific curriculum content (e.g., specific stressors, family rejection, safety nets). Participants expressed strong interest in the financial components, whereas facilitators noted opportunities to expand content addressing LGBTQ+‐specific financial challenges (e.g., healthcare, insurance barriers, lack of family support, and family planning). These findings align with structural gaps in LGBTQ+‐tailored financial resources (Badgett et al. 2021) and suggest that integrating financial and relationship content is both well received and responsive to participants' needs. Participants and facilitators also recommended more inclusion of diverse family structures (e.g., CNM relationships), support systems, and neurodivergent experiences. The request for content about CNM relationships highlights limitations in current CRE models, which typically assume monogamous, dyadic partnerships, and may not reflect the full range of relational experiences (Scott et al. 2019; Gupta et al. 2024). Together, these findings underscore the importance of intersectional adaptations to better address overlapping forms of marginalization. Implementation strengths, including cultural competency training and a team‐based facilitation approach likely enhanced staff preparedness and contributed to a safe, affirming environment (Yu et al. 2023), even where LGBTQ+‐specific content could be further strengthened.

Regarding feasibility, findings supported the implementation of LGBTQ+ TOGETHER, with some constraints. Participants who remained in the program demonstrated strong engagement and high attendance, although attrition was notable early in the intervention (25%), with several participants discontinuing after one or two sessions. This pattern is consistent with prior CREs (Williamson et al. 2023), though qualitative findings provide important nuances. Reasons for early dropout included participants mistaking the program for therapy, scheduling challenges despite evening and weekend options, and family emergencies. Notably, even brief, non‐affirming elements may undermine engagement, as one participant dropped out due to reported concerns about the inclusivity of funding agency required surveys and perceived a lack of LGBTQ+‐centered content during the first session. These findings point to the importance of clear program framing during recruitment, flexible delivery options, and ensuring that all aspects of the interventions are affirming and inclusive. Despite these challenges, feasibility was supported by program strengths. Participants and staff valued the LGBTQ+‐specific format for fostering safety through community connectedness (Frost et al. 2017), suggesting that such formats may enhance experiences, enrollment, and retention. Feasibility was further supported by the program's intentional design and implementation strategies, including compensated time for facilitator preparation, coordination among staff, ongoing supervision and cross‐team consultation, and pre‐implementation preparation (e.g., cultural competency training, curriculum updates), which promoted fidelity and responsiveness. Iterative, reflexive adjustments during the pilot phase further supported implementation (Stirman 2022).

At the same time, feasibility was constrained by workforce limitations, particularly challenges in recruiting a diverse pool of LGBTQ+‐identified facilitators, which affected implementation flexibility (e.g., coverage when facilitators were unavailable). These challenges reflect broader workforce constraints, particularly in recruiting LGBTQ+ financial counselors. As of March 1, 2026, less than 0.1% of certified financial planning professionals (CFPs) identified as non‐binary, and other sexual orientation and gender identity statistics were not reported (Certified Financial Planner Board 2026). Ethnic and racial diversity among facilitators was also identified as important by participants and facilitators. This limited representation is reflected in the broader field; for example, among US CFPs, 81.4% identify as White (Certified Financial Planner Board 2026). These patterns underscore systemic constraints that limit the feasibility of culturally responsive adaptations that aim to reflect diverse identities.

Throughout, differences emerged in participant and staff experience. Participant and case manager feedback was generally supportive, whereas facilitators offered more critical feedback and emphasized areas for improvement. This difference may reflect facilitators' professional roles, deeper engagement with program content, greater lived and professional insight into LGBTQ+ experiences, and prior exposure to the non‐adapted version of the program. For instance, participants found the curriculum helpful and relevant, yet facilitators frequently highlighted limitations in LGBTQ+‐specific content, and noted it was not originally designed for LGBTQ+ populations. This distinction has important implications for CRE design. Facilitator cultural competence and LGBTQ+ content and group composition may enhance relevance and engagement, but simultaneously mask gaps in curricular content that participants may not fully articulate. This tension reflects broader tradeoffs in intervention design, where adaptations may support feasibility but may not fully capture population‐specific needs. These findings point to a broader question in the CRE literature regarding when adaptation of existing evidence‐based programs is sufficient versus when population‐specific program development may be warranted (Chen et al. 2013). Programs developed specifically for LGBTQ+ populations may offer greater cultural specificity but require substantial resources for development, testing, and scaling (Aarons et al. 2017). Given that only a few CREs have been developed specifically for–rather than adapted for–LGBTQ+ populations (Whitton et al. 2017; Macapagal et al. 2019; Rostosky et al. 2025), future research should examine differences in engagement, perceived relevance, and outcomes between adapted and population‐specific models. These findings underscore the importance of incorporating both participant and staff perspectives when evaluating adaptations, particularly in early‐stage or pilot studies.

### 4.1 Limitations

This pilot study included a relatively small sample of LGBTQ+ individuals in one US metropolitan region, limiting generalizability to other geographic regions. Limited demographic information was available for some participants, particularly staff, which restricted our ability to fully characterize the sample and examine how characteristics such as parenthood or other sociodemographic factors may have shaped program experiences and perspectives. Although TGD participants were well represented, demographic information about neurodivergent and CNM identities was not collected, which may obscure differences in program experiences. Additionally, limited participation from participants who did not complete the program restricted our ability to understand reasons for attrition and whether they were program‐related. Some adaptations requiring identity‐matched facilitators may be constrained by workforce availability, and facilitators without shared identities must be prepared to work competently across diverse groups. Additionally, some facilitators held multiple roles within the program (e.g., as trainees and staff), which may have influenced their perspective and willingness to provide critical feedback. Because curriculum adherence was self‐reported by facilitators, findings may be subject to recall and social desirability biases. Given that quantitative analyses were limited to descriptive statistics, findings should therefore be interpreted as preliminary evidence of implementation success and replicated in future research.

## 5 Implications and Recommendations

This pilot study highlights the need to continue adapting relationship and financial education programs to better serve LGBTQ+ populations. Future research should evaluate the effectiveness of the adaptation using more rigorous study designs and advanced analytic analyses and examine similar adaptation across diverse geographic regions and contexts. Studies should also explicitly incorporate the experiences of and content addressing CNM relationships and neurodivergent participants, as these experiences are disproportionately common among LGBTQ+ populations (Gupta et al. 2024; Strang et al. 2014).

Given participants' emphasis on safe, affirming environments and the value of LGBTQ+‐identified facilitation, programs delivering relationship and financial education should consider using similar multi‐component adaptation models to enhance cultural competence, inclusivity, and responsiveness to diverse family structures. Initial steps may include integrating inclusive language and examples into curriculum materials, developing referral networks that connect participants to affirming resources, and prioritizing recruitment and retention of staff who reflect the identities of the population served. Programs should also provide ongoing cultural competency training focused on communities served, and in the case of LGBTQ+ programs, training should include content on TGD, CNM, and neurodivergent identities.

Given the structural and workforce constraints identified, funding bodies, training programs, and professional organizations play a critical role in sustaining this work by prioritizing strategies to recruit, train, and retain a diverse workforce, particularly with respect to LGBTQ+ and BIPOC professionals. Expanding workforce diversity, especially among financial planners, may help reduce structural barriers to relational and financial wellbeing for LGBTQ+ people. Overall findings from this study suggest that culturally responsive approaches in CREs are not only acceptable, appropriate, and feasible, but necessary to promote equity.

## 6 Conclusion

LGBTQ+ TOGETHER was found to be acceptable, appropriate, and feasible in meeting the needs of LGBTQ+ participants. Findings from survey and qualitative data suggest that these adaptations contributed to an affirming and engaging program environment and relevant content that supported participant trust, while also identifying areas for continued refinement. Results highlight the need for deeper, intersectional, and structural adaptations to ensure that program content more fully reflects the diverse and overlapping identities and experiences of LGBTQ+ individuals. Importantly, these findings emphasize the value of culturally tailored adaptations for engagement and relevance in CREs for LGBTQ+ populations. Future research should examine this model across larger and more diverse samples and settings to assess scalability and long‐term outcomes. More broadly, this work contributes to cultural adaptation and implementation science by demonstrating how evidence‐based programs can be modified to improve fit, engagement, and equity in underserved populations.

## Supporting information


Supporting File

## Data Availability

The data that support the findings of this study are available on request from the corresponding author. The data are not publicly available due to privacy or ethical restrictions.
