# Supplementary material for: Pilot Test of the Relationship and Financial Education TOGETHER Program for LGBTQ+ People in Relationships
Source: J Marital Fam Ther. 2026 Oct 1;52(4):e70176. doi: 10.1111/jmft.70176 (PMC13628280; doi:10.1111/jmft.70176)
Supplement: Supplementary file 1 [file JMFT-52-0-s001.docx]

**Supplemental Materials**

| **Table SI**  *Interview and Focus Group Questions* | |
| --- | --- |
| Participant Focus Group Questions | 1. How did you first hear about the TOGETHER program? 2. What made you want to enroll in the TOGETHER program? 3. What was your impression of the advertising materials for TOGETHER? 4. What were your overall impressions of the program? 5. Would you recommend this program to your LGBTQ+ friends or family? Why or why not? 6. Did anything in particular make you and/or your partner feel comfortable/welcomed while participating in the sessions? 7. Was there anything particularly uncomfortable about participating in the sessions? 8. In what ways did you feel included and safe during the sessions? 9. What was your overall impression of the TOGETHER facilitators and staff’s awareness of and respect for people with LGBTQ+ identities? 10. In what ways did you think the sessions were relevant to your life experiences? 11. What types of resources would have been helpful to you during or after the workshop to better meet the needs of LGBTQ+ participants? |
| Case Managers Focus Group Questions | 1. What is your perspective on the overall effectiveness of the TOGETHER program in connecting LGBTQ+ participants to community resources/services? 2. What strengths and challenges did you have as a case manager in working with LGBTQ+ participants? 3. In what ways do you think the trainings prepared you to work with/deliver services to LGBTQ+ participants? 4. In what ways does the program make the workshop a safe and inclusive environment for LGBTQ+ participants? 5. What is your experience with screening the appropriateness of community resources for TOGETHER program LGBTQ+ participants? 6. What have you noticed about the needs assessment of LGBTQ+ participants you have worked with? 7. How has the individual and partner development plan been received by the participants? 8. What community resources do you think best relate to LGBTQ+ participants? 9. In what ways do your identities impact your work with LGBTQ+ participants? 10. Is there anything else you’d like to share about your experience working with TOGETHER program LGBTQ+ participants? |
| Facilitator Interview Questions | 1. What is your perspective on the overall effectiveness of the TOGETHER program in meeting the needs of LGBTQ+ participants in regards to improving their communication, stress management, problem solving, and financial management knowledge and skills? 2. What strengths and challenges did you have as a facilitator in delivering content to LGBTQ+ participants? 3. In what ways do you think the program prepared you to deliver the curriculum for LGBTQ+ participants? 4. In what ways does the program make the workshop a safe and inclusive environment for LGBTQ+ participants? 5. In what ways did you attempt to make the workshop a safe and inclusive environment for LGBTQ+ participants? 6. What TOGETHER program content do you think best relates to LGBTQ+ participants? 7. What other topics related to relationship and financial education for LGBTQ+ participants should be included in the program’s sessions, homework, or other? 8. In what ways do your identities impact your facilitation of groups with LGBTQ+ participants? 9. Is there anything else you’d like to share about your experience delivering the TOGETHER curriculum for LGBTQ+ participants? 10. Is there anything else you’d like to share about the appropriateness of the TOGETHER curriculum for LGBTQ+ participants? |
